# Edging along a Warming Coast: A Range Extension for a Common Sandy Beach Crab

**DOI:** 10.1371/journal.pone.0141976

**Published:** 2015-11-02

**Authors:** David S. Schoeman, Thomas A. Schlacher, Alan R. Jones, Anna Murray, Chantal M. Huijbers, Andrew D. Olds, Rod M. Connolly

**Affiliations:** 1 School of Science & Engineering, University of the Sunshine Coast, Maroochydore DC, Queensland 4558, Australia; 2 Australian Museum Research Institute, 6 College St, Sydney, New South Wales 2010, Australia; 3 Australian Rivers Institute–Coast & Estuaries, and School of Environment, Griffith University, Gold Coast, Queensland 4222, Australia; University of Western Sydney, AUSTRALIA

## Abstract

Determining the position of range edges is the first step in developing an understanding of the ecological and evolutionary dynamics in play as species’ ranges shift in response to climate change. Here, we study the leading (poleward) range edge of *Ocypode cordimanus*, a ghost crab that is common along the central to northern east coast of Australia. Our study establishes the poleward range edge of adults of this species to be at Merimbula (36.90°S, 149.93°E), 270 km (along the coast) south of the previous southernmost museum record. We also establish that dispersal of pelagic larvae results in recruitment to beaches 248 km (along the coast; 0.9° of latitude) beyond the adult range edge we have documented here. Although we cannot conclusively demonstrate that the leading range edge for this species has moved polewards in response to climate change, this range edge does fall within a “hotspot” of ocean warming, where surface isotherms are moving southwards along the coast at 20–50 km.decade^-1^; coastal air temperatures in the region are also warming. If these patterns persist, future range extensions could be anticipated. On the basis of their ecology, allied with their occupancy of ocean beaches, which are home to taxa that are particularly amenable to climate-change studies, we propose that ghost crabs like *O*. *cordimanus* represent ideal model organisms with which to study ecological and evolutionary processes associated with climate change. The fact that “hotspots” of ocean warming on four other continents correspond with poleward range edges of ghost crab species suggests that results of hypothesis tests could be generalized, yielding excellent opportunities to rapidly progress knowledge in this field.

## Introduction

At its essence, ecology strives to explain why organisms are where they are [1,2]. The quest for answers to this question has a long history (e.g., [3,4]), but never has it been more important [5,6]: climate change and anthropogenic relocations are combining to reshuffle species assemblages globally [6–12]. An understanding of the rate and magnitude of these modifications to species’ distributions has become important for planning conservation interventions and managing invasive species [10,12–16], and for securing food resources into the future [17–20].

The first stage of understanding range shifts is quantifying the distributional limits of individual species. These limits, commonly referred to as range edges, are determined by interactions between geographic features of the land/seascape (including habitat availability), climate, life-history traits and demographics, competitive or facilitative biotic interactions, physiological limits, evolutionary processes, and, on occasion, serendipity [2,21–23]. The abundance of a species and its population stability are usually lower near its range edge [24]. Whilst isolated individuals, or even aggregations of recruits, might sporadically occur beyond the range edge, their abundance inevitably declines to zero because the rate of mortality and/or emigration exceeds the rate of recruitment and/or immigration [2,24].

Because species may have relatively wide distributions across landscapes, they can exist in isolated population fragments, and because there are often difficulties in detecting presence, or confirming absence of many species, there are very few species for which range edges can be determined with high precision and accuracy [25,26]. Mapping species distributions is therefore usually a probabilistic exercise in numerical modelling [21,27]. An exception to this generalization is provided by one of the most ubiquitous inhabitants of many sandy beaches around the world: ghost crabs.

Ghost crabs (family Ocypodidae, subfamily Ocypodinae Rafinesque 1815) occupy the upper shores of ocean-exposed sandy beaches from the tropics to temperate latitudes, with several species having large geographic ranges that span many degrees of latitude [28]. Importantly, ghost crabs are among the few species to construct semi-permanent burrows on beaches. The openings of occupied ghost-crab burrows are readily distinguished from other holes in the beach by their characteristic shape, fans of sand that result from burrow maintenance, and crab tracks caused by regular excursions in search of food. Because adult ghost crabs are fossorial inside their burrows during the day (at least in the presence of humans) [29], they can be easily collected by simple excavation of burrows when species identification is required. Although individual crabs might excavate multiple burrows, burrow counts are, with a few caveats, a reliable index of population size [30,31]. In combination, these features mean that relatively little effort is required to determine the geographical limits of ghost crab populations with reasonable precision and confidence.

While range limits can be determined with some certainty for other marine organisms, including corals, seagrasses and kelps, among others, these tend either to have more restrictive environmental requirements, or to be less accessible than beaches over long stretches of coastline. By the same token, other shoreline invertebrates tend either to be difficult to detect, especially at low densities, as is the case for most rocky shore species, or to occupy habitats that are more fragmented (along the shore) than beaches, as is the case for fiddler crabs in estuaries.

On this basis, we studied the position of the poleward range edge of the distribution of the ghost crab *Ocypode cordimanus* Latreille, 1818, on the Australian east coast. This species has a wide distribution across the Indo-West Pacific [32], and has been recorded in Australia between the Kimberley (northern Western Australia) and the central coast of New South Wales [33,34]. Over most of this range, *O*. *cordimanus* is sympatric with the larger *O*. *ceratophthalma* (Pallas, 1772), as it is throughout the Indo-Pacific [34]. We worked on the east coast of Australia because it is aligned north-south and spans a wide range in both sea and air temperatures. This region has also experienced rapid ocean warming, especially in the vicinity of the putative poleward range edge of the species of interest [35], with isotherms of sea-surface temperature moving southwards along the coast at a rate of 20–50 km.decade^-1^ [36,37]. By contrast, corresponding warming of coastal air temperatures is more moderate in a global context [37,38].

We aimed to determine the position and short-term temporal stability of the range edge of *O*. *cordimanus*, and to explore the utility of this species as a model organism for understanding range-edge dynamics. Specifically, we predicted that (1) we would be able to reliably detect the position of the leading range edge of *O*. *cordimanus*, and that (2) in accordance with warming trends, this position would be further south than that of previous records.

## Materials and Methods

Ethics Statement: Our target species, *Ocypode cordimanus*, is not listed as threatened or protected anywhere in Australia. Sampling in New South Wales was facilitated by New South Wales Department of Primary Industries Scientific Collection Permit number F86/2163-6.3, on which ARJ and AM are named collectors. Since only juvenile crabs were present in Victoria, no Scientific License was required by the Victorian Department of Environment and Primary Industries (http://www.depi.vic.gov.au/agriculture-and-food/animal-health-and-welfare/animals-used-in-research-and-teaching/licensing/activities-requiring-a-license). Although work with adult crabs requires ethics clearance in Victoria, work with juveniles does not; there are no specific ethics requirements for working with invertebrates in New South Wales. All reasonable efforts were made to minimize disturbance to native wildlife. With the exception of the specimens lodged with the Australian Museum, all ghost crabs excavated were returned immediately, and unharmed, to the beach. In no instance was research undertaken on private land. In accordance with our permit conditions, local and/or regional conservation and management authorities were telephoned in advance of our research to notify them of our intended presence and the nature of our work. No other permissions were necessary for our research.

**Fig 1:**
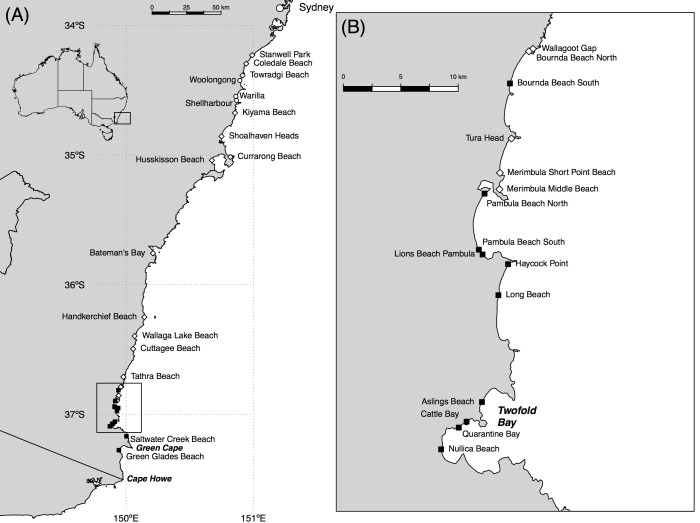
The distribution of adult *Ocypode cordimanus*. (A) along the central and south coast of New South Wales (NSW), Australia, as determined by detailed observation in January and February 2013. Inset provides spatial context at the continental scale. (B) Finer resolution sampling was undertaken in the vicinity of the range edge; geographic context provided by the box in (A). White diamonds indicate confirmed presence of the species; black squares indicate confirmed absence. Cities and towns mentioned in the manuscript are indicated by white circles; significant coastal features are labeled in italics. Underlying data are available in S1 Table; coastline derived by modification of a shapefile sourced from Geoscience Australia (https://data.gov.au/dataset/geodata-coast-100k-2004).

Expectations of the position of the poleward range edge of ghost crabs were provided by a review of the literature, consultation of the Atlas of Living Australia (http://www.ala.org.au), and detailed analysis of records held by two museums: the Australian Museum, Sydney, and Museum Victoria, Melbourne. These sources all pointed to a range edge in the vicinity of Wollongong, central New South Wales. We therefore undertook an exploratory survey in late January and early February 2013 that extended from Stanwell Park, 22 km north of Wollongong, southwards to Green Glades, in the lee of Green Cape, in the extreme southeast of New South Wales (Fig 1; S1 Table). Sample sites were initially widely spaced (10–40 km apart), with increasingly fine resolution (determined iteratively by presence/absence of ghost crabs) as we focused in on the range edge (Fig 1).

Teams of two (and occasionally three) experienced beach ecologists searched the upper shore and foredunes of each sampled beach for signs of ghost-crab burrows, paying particular attention to areas around carrion, logs, vegetation and scarps in the beach profile, where ghost crabs are known to aggregate [28,39]. We focused on detecting larger burrows (> 20 mm), as these belonged to specimens that had survived at least one winter and hence could be considered to represent a population with at least short-term persistence at a site. This focus on larger burrows is important because annual recruitment events might occur beyond the adult range edge [40,41].

At most beaches (including all beaches sampled within 20 km of the range edge), a haphazard selection of burrows was excavated by hand to confirm the species identity of resident crabs. A small number of specimens was collected from some beaches at the range edge and deposited in the Australian Museum Marine Invertebrates Collection. Where ghost crab burrows were not detected, we searched a minimum of 1 km (alongshore) of beach, with the sampling team traversing both the upper and lower shores of such beaches; for beaches shorter than 1 km, we searched the entire length of the beach. No attempt was made to estimate ghost crab population size, or to quantify population size structure, because our aim was purely to determine the presence of crabs that had survived the previous winter.

**Fig 2:**
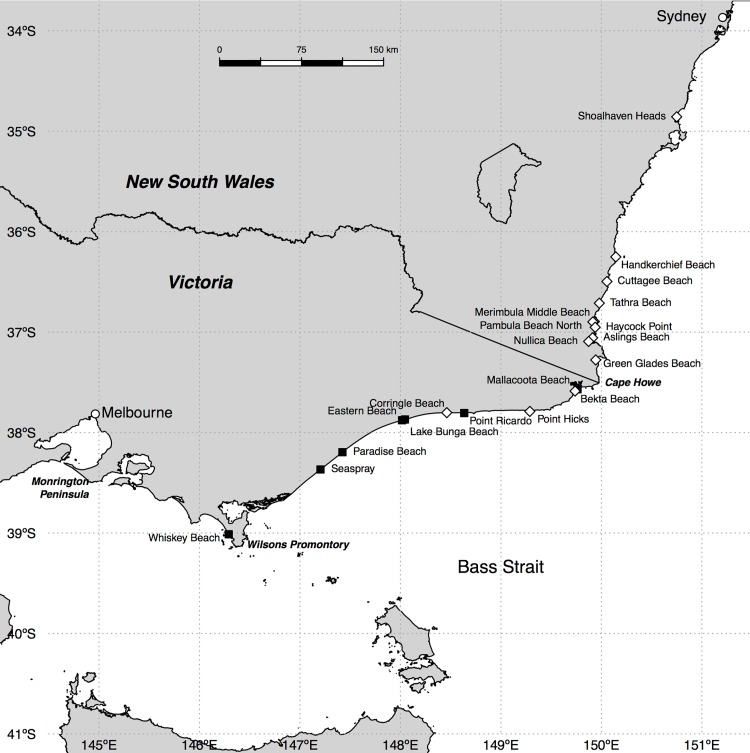
The distribution of *Ocypode cordimanus* recruits (young of the year) in May 2013. White diamonds indicate confirmed presence; black squares indicate confirmed absence. Underlying data are available in S1 Table; coastline derived by modification of a shapefile sourced from Geoscience Australia (https://data.gov.au/dataset/geodata-coast-100k-2004).

A similar approach was used in May, June and October 2013, first to confirm the position of the range edge of adult distribution established earlier in the year, and second to establish the area over which juvenile ghost crabs had recruited. Because we aimed to determine whether recruitment occurred beyond the adult range (May), we extended our surveys to the southwest as far as Wilson’s Promontory on the Bass Strait, the southernmost point on the Australian mainland (Fig 2; S1 Table).

The beaches we inspected (Figs 1 and 2) ranged in length (between rocky headlands, estuaries, or other interruptions) from < 1 km to > 100 km, were generally wave-dominated and of the intermediate morphodynamic state, experienced a maximum tide range of around 2 m, and comprised mainly (> 80%) quartz-rich sediments [42,43]. The longest stretch of largely uninterrupted rocky coastline in the study area (~ 32 km) was around Green Cape. This rocky area does contain several short (< 0.5 km) pocket beaches, but these are not readily accessible, and hence could not be sampled.

To study recent trends in annual temperatures, we used gridded data products for monthly mean sea (Hadley Centre Sea Ice and Sea Surface Temperature data set, HadISST1.1, 1° grid) and air (Climate Research Unit, University of East Anglia, CRU TS3.22, 0.5° grid) temperatures, globally [44,45]. To quantify spatial patterns, we report the arithmetic mean temperature per grid cell for the 25 years ending in 2013; for temporal trends over the corresponding period, we compute for each grid cell the slope of the simple linear regression of annual mean temperature against year (i.e., the rate of temperature change through time).

## Results

Australian Museum records indicate that the southernmost confirmed specimen of *Ocypode cordimanus* was collected from Warilla Beach (34.55°S, 150.87°E) in 2011, while the southernmost recorded specimens of *Ocypode ceratophthalma* were collected slightly further south at Shellharbour (34.58°S, 150.87°E) in 1923. The southernmost Australian Museum record of *Ocypode* sp. is from northern end of Tathra Beach (36.70°S, 149.98°E), some 236 km south of Shellharbour. This record, collected in 2008, comprises two small juveniles of 6–8 mm carapace length, which are too small to identify to species level. In addition to these confirmed museum records, a reliable observer (Shane Ahyong, Australian Museum) reported sighting *O*. *cordimanus* over the past few decades on south-coast beaches, including Tathra and Merimbula beaches in January 1993, but no information is available about the size of these specimens or the persistence of their populations. Together, these observations inform the distribution maps of Sakai and Türkay [34], which place the poleward range edge for *O*. *cordimanus* in southeast Australia in south-central New South Wales.

During January and February 2013, we excavated several ghost crab burrows on each beach from Currarong Beach southwards, but found no specimens of *O*. *ceratophthalma*, and observed no burrows large enough to suggest the presence of adults of this larger species, which is sympatric with *O*. *cordimanus* from Wollongong northwards [28]. We therefore conclude that the poleward range edge of *O*. *ceratophthalma* is most likely between Currarong and Wollongong, and that we can discount the presence of this species on the beaches we sampled further south.

By contrast, we found sizeable populations of adult *O*. *cordimanus* on all beaches we visited from Tathra Beach (36.73°S, 149.99°E) northward, and smaller populations on beaches up to 20 km to the south of Tathra Beach, largely in the sheltered lee of rocky headlands (Fig 1). The only exception to this was the southern end of the beach at Bournda, at which no ghost crabs or burrows were detected. The southernmost position at which adult *O*. *cordimanus* were found was Middle Beach, Merimbula (36.90°S, 149.93°E). No ghost crabs or holes of dimension attributable to adult ghost crabs were detected south of this location (Fig 1), although isolated instances of small (≤ 10 mm diameter) unoccupied burrows, possibly dug by recently settled ghost crab post-larvae, were detected at Aslings and Quarantine Bay beaches (Fig 1).

During May 2013, ghost crab recruits were present on all beaches visited (Fig 2) north of Point Ricardo, Victoria (37.81°S, 148.64°E). Note that although Corringle Beach (37.80°S, 148.46°E) is west of Point Ricardo, the orientation of the coastline means that it is also slightly to the north. The only exception to this pattern was Mallacoota Beach (Fig 2), but this was probably because there was access only to the sand spit to the west of the Mallacoota Inlet, which is essentially a flood-tide delta that is vulnerable to overtopping during heavy rainfall and therefore is not ideal ghost crab habitat. These observations confirm that ghost crab larvae can move southwards around Green Cape and Cape Howe to reach the eastern Victorian coastline. However, during this survey, no ghost crab burrows large enough to be inhabited by adults were found south of Middle Beach, Merimbula, confirming this as the southern range edge of the adult population prior to the winter of 2013.

Follow-up surveys in June and October 2013 reinforced our previous findings. Our observations therefore extend the formally recorded range for *O*. *cordimanus* 270 km along the coast (2.35°, ~ 262 km southwards), and confirm that this species can survive the winter as far south as Merimbula.

**Fig 3:**
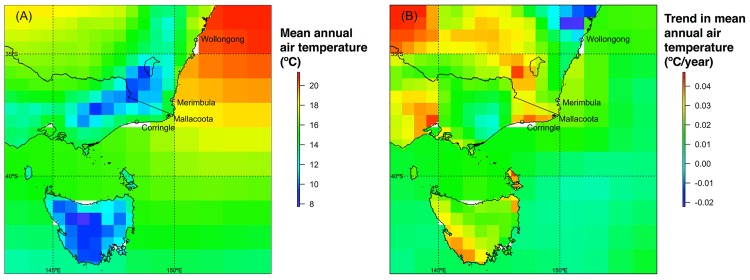
Coastal air (CRU TS3.22; 0.5° grid) and seawater (HadISST1.1; 1° grid) temperature trends over the 25-year period 1989–2013, inclusive. (A) Mean annual temperatures, and (B) temporal trends in temperature change, as indicated by the slope of a simple linear regression of mean annual temperature as a function of time. Temperature grids were clipped to the coast at a resolution of 0.1°; unfilled partial grid squares evident at various points along the coast had no corresponding sea-surface temperatures, but may be assumed to have similar temperatures/trends as adjacent cells. Coastline derived by modification of a shapefile sourced from Geoscience Australia (https://data.gov.au/dataset/geodata-coast-100k-2004).

Along the Australian southeast coast, observed temperature patterns confirm the presumed spatial gradient in air and seawater temperatures from north to south (Fig 3A). However, temporal trends in temperatures are variable (Fig 3B), ranging from slight cooling for coastal air temperatures along the central coast of New South Wales, to strong warming of ocean and coastal air temperatures in southern New South Wales and extending into the Bass Strait.

## Discussion

Our observations confirmed the first of our *a priori* hypotheses, and provided strong support for the second. Specifically, we established that the poleward range edge for adult *O*. *cordimanus* is located at Merimbula’s Middle Beach (36.90°S, 149.99°E). This range edge was stable over at least two winters, with crabs readily detected there throughout the sampling period, but never being found, as adults, further south. Second, we extended the formally recorded range edge for adult *O*. *cordimanus* populations by 270 km (along the coast; ~ 262 km north-south; 2.35° latitude) southward along the eastern Australian coast. Unfortunately, previous distributional information relies on museum collections and anecdotal evidence. The caveats associated with using such presence-only historical data to infer range expansions are well known [26]. Moreover, although anecdotal observations of ghost crabs have previously been made in the vicinity of our southernmost record, they provide no information on whether the crabs observed were young of the year or whether they were members of a persistent population. We therefore cannot conclusively discount the possibility that isolated individuals have previously occupied beaches in the vicinity of the range edge established here, although the lack of quantitative observations in this regard renders unlikely the presence of persistent adult populations.

Our observations nevertheless demonstrated that juvenile ghost crabs extend seasonally at least 260 km (along the coast; 1.1° of latitude) beyond their previously recorded range edge of Tathra Beach (36.70°S 149.98°E), and 248 km (along the coast; 0.9° of latitude) beyond the adult range edge we established, recruiting to beaches as far west as Corringle Beach (37.80°S 148.46°E). This suggests that range extensions of *O*. *cordimanus* populations, like those of other *Ocypode* species [40,41], are unlikely to be due to recruitment limitations under present conditions.

We formally establish a stable poleward range edge for *O*. *cordimanus* at Merimbula, and demonstrate that the position of this range edge is not determined primarily by recruitment limitation under present conditions. Together these findings have important implications for interpreting the consequences of warming on coastal species in the region. Our analysis of global temperature datasets confirms that while both air and ocean temperatures decrease from north to south along the coast, there is a stronger spatial gradient in mean seawater than air temperatures. These analyses also show that spatial patterns in the rate of temperature change are more complex on land than they are in the ocean. Specifically, while our results reiterate the identification of the Australian southeast coast as a recent hotspot of ocean warming [35], they also confirm that warming of coastal air temperatures in the region has been only intermediate in a global context [37,38]. Nevertheless, the most rapid warming of coastal air temperatures, locally, was observed immediately to the south of the *O*. *cordimanus* range edge at Merimbula, extending westwards along the northern shore of the Bass Strait. Both air and seawater temperatures in the region are expected to warm at similar or faster rates into the future [38,46].

Observations that the distributional ranges of rocky intertidal fauna of Tasmania (to the south of our study area) are moving southwards at rates of 20–250 km.decade^-1^ [47], correspond with estimated rates and directions of isotherm movements in coastal waters; by contrast isotherms of coastal surface-air temperatures are moving considerably more slowly in this region [36,37]. This raises the question of which temperature variable, or combination of variables, regulates the position of the range edge for *O*. *cordimanus*. Since this species overwinters in burrows in the supralittoral zone [28], beyond the reach of waves from all but the most intense of storms, it is likely that sediment temperatures will be most relevant.

Available evidence suggests that supralittoral sediment temperatures at a depth of 50 cm (roughly the depth of ghost crab burrows [28]) are mediated by both air and seawater temperatures [48]. It should be noted, however, that this generalization pertains to summer temperatures and tropical beaches. Under such circumstances, air temperatures are likely warmer than ocean temperatures. During winter at the higher latitudes of our study, however, seawater temperatures would likely be warmer than minimum air temperatures, so might play an increasingly important role in mediating sub-surface sediment temperatures. Although this remains an open question for future research, the fact that both air and seawater temperatures south of the range edge are warming rapidly suggests that range expansion is likely, especially since recruitment is not presently a limiting factor.

Our findings might be more significant because of the presence of a biogeographic boundary in the vicinity of Merimbula. For example, Waters et al. [49] placed Twofold Bay, 20 km south of Merimbula, at the eastern edge of the Maugean (cool, southern) Province on the basis of macroalgal community structure. Barnacle communities largely corroborate this biogeographic break [50]. Moreover, there is significant overlap between the Maugean and the adjacent Peronian (warmer, southeastern) Provinces between Twofold Bay and the Mornington Peninsula, which forms the eastern head of Port Philip Bay, suggesting that this is an area of considerable turnover in species pools. Because the large-scale distribution of beach macroinfaunal species tends to be restricted less by biogeographic boundaries than is that of rocky shore species [51], we would anticipate that a poleward-edge range extension of *O*. *cordimanus* might provide an early indication of broader-scale ecological change to follow.

Interestingly, the overlap between the Peronian and Maugean Provinces extends not only along the northern shores of the Bass Strait, but also along the northern and eastern seaboards of Tasmania. Although ghost crabs have not been observed along the Tasmanian coast, and extant macroalgal communities there are more consistent with those from the cooler Maugean Province than with those from the warmer Peronian Province [49], it seems plausible that climate change might facilitate the movement of *O*. *cordimanus* across Bass Straight and into this region, via the East Australian Current Extension. The recruitment of this crab species to beaches along the northern shore of the Bass Strait (the Victorian east coast), almost 250 km (measured along the coast) from the southern edge of the adult distribution, suggests that if *O*. *cordimanus* establishes adult populations there, its larvae might in future be capable of surviving long enough to cross the Strait (possibly using islands as inter-generational stepping stones). Moreover, several other intertidal species are known to have extended their ranges southwards along the Tasmanian coast [47,52,53], sometimes with adverse ecological effects [54,55]. Thus, if ghost crabs were to arrive in northern Tasmania in the future, it is plausible that they would continue to extend their range southwards.

The detection and attribution of climate-change impacts requires the collection of long and robust time series of observational data [15,56–58]. Because this is often a costly and time-consuming exercise, accessible and easily identifiable taxa should be targeted for such studies. Not only are beaches amongst the most accessible of coastal habitats, they are also useful model systems in which to test hypotheses regarding climate-change impacts [59]. Of the fauna found on beaches, ghost crabs appear to be ideal model organisms for such studies: they occur widely along tropical and warm-temperate coastlines around the world [28], where they are well-known by the public and scientists alike; their presence or absence is relatively easy to determine, at least for part of the year; although they are sensitive to some human impacts [60,61], they are seldom eliminated by human use of the shore, except *in extremis*; and they are also relatively easy to maintain in the laboratory, making them amenable to experimentation.

Here we have proposed that ghost crabs are model indicators of climate change in the coastal zone. Simple predictions can be articulated, which, if upheld, would confirm our hypothesis. First, the poleward range edge of ghost crab populations should fluctuate in position from year to year as a function of prevailing temperatures, either of air, water, or of the sediment at the depth of crab burrows. Second, a clear mechanism will exist for such temperature control. Here we suggest a simple conceptual model based on the biology of the taxon [28]: larval dispersion and subsequent recruitment polewards of the adult range edge during spring and summer (as documented here) regularly establishes potential crab populations beyond the existing range edge; whether these potential populations persist depends on whether individual crabs can survive the winter [40]. If this mechanism holds, range extensions should match the rate of migration of isotherms associated with minimum winter temperatures corresponding to thresholds of ghost-crab survival. Alternatively, if range extensions do not match the rate of isotherm migration, the underlying reasons should be detectable in physiological or behavioral plasticity, and be reflected by population genetics.

**Fig 4:**
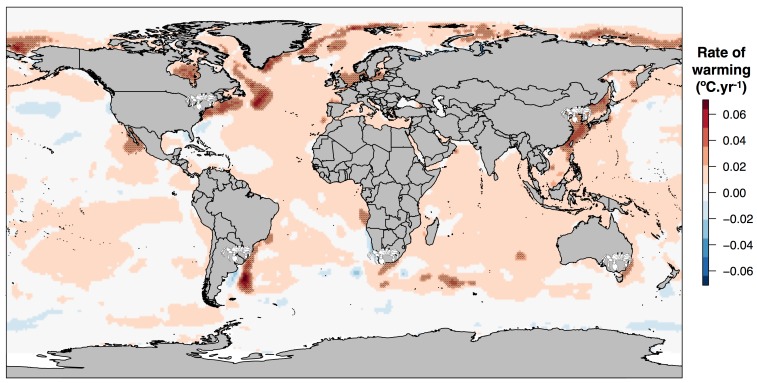
Ghost crab poleward range edges in relation to ocean warming. Rates of ocean warming (°C.year^-1^) from linear regression of annually averaged Hadley Centre HadISST 1.1 data (see Methods) for the 50-year period 1961–2010. Light hatching indicates areas in the upper 5^th^ percentile of ocean warming, globally. Crab symbols indicate positions of ghost-crab poleward range edges (extracted from Lucrezi and Schlacher [28] and indicated approximately by the smaller left claw of the symbolic crab) that correspond with these ocean-warming “hotspots”. Coastline derived from shapefile available at http://thematicmapping.org/downloads/world_borders.php.

Importantly, there is an opportunity to generalize findings for such hypotheses because four other hotspots of ocean warming (Fig 4; [46]) coincide with known poleward range edges of different ghost crab species [28]: South Africa (*O*. *madagascariensis* and *O*. *ryderii*); Uruguay (*O*. *quadrata*); the eastern seaboard of the United States (*O*. *quadrata*); and Japan/South Korea (*O*. *stimpsoni*). In all instances, except Uruguay, the area of rapid warming extends polewards of the ghost crab range edge. In the latter case, a pool of cooling water is located south of the Rio De La Plata, suggesting that further range extension should be blocked. This provides a useful large-scale test of the hypothesized mechanism underlying the range-edge dynamics.

## Supporting Information

S1 Table The positions of beaches sampled over the duration of the studty, with notes on the presence (1) or absence (0) of *Ocypode cordimanus* specimens. In each instance, presence and absence of adult (>20 mm diameter) holes was noted. In addition, presence and absence of recruits was noted during May 2013. Because we did not retain detailed records for recruits on other trips, these are recorded as NA.
(PDF)
