# Supplementary material for: Edging along a Warming Coast: A Range Extension for a Common Sandy Beach Crab
Source: PLoS One. 2015 Nov 2;10(11):e0141976. doi: 10.1371/journal.pone.0141976 (PMC4629900; doi:10.1371/journal.pone.0141976)
Supplement: S1 Table — In each instance, presence and absence of adult (>20 mm diameter) holes was noted. In addition, presence and absence of recruits was noted during May 2013. Because we did not retain detailed records for recruits on other trips, these are recorded as NA. (PDF) [file pone.0141976.s001.pdf]

S1 Table: The positions of beaches sampled over the duration of the studty, with notes on the presence (1) or absence (0) of *Ocypode cordimanus* specimens. In each instance, presence and absence of adult (>20 mm diameter) holes was noted. In addition, presence and absence of recruits was noted during May 2013. Because we did not retain detailed records for recruits on other trips, these are recorded as NA.

Name	Lat	Lon	Date	Adult	Recruit
Currarong Beach	-35.0152	150.8189	29/01/13	1	NA
Shoalhaven Heads	-34.8563	150.7494	29/01/13	1	NA
Kiyama Beach	-34.6756	150.8550	29/01/13	1	NA
Towradgi Beach	-34.3861	150.9145	29/01/13	1	NA
Coledale Beach	-34.2953	150.9432	29/01/13	1	NA
Stanwell Park	-34.2303	150.9900	29/01/13	1	NA
Tathra Beach	-36.7118	149.9789	30/01/13	1	NA
Cuttagee Beach	-36.4959	150.0552	30/01/13	1	NA
Wallaga Lake Beach	-36.3964	150.0676	30/01/13	1	NA
Handkerchief Beach	-36.2509	150.1435	30/01/13	1	NA
Bateman's Bay	-35.7579	150.2114	30/01/13	1	NA
Husskisson Beach	-35.0405	150.6737	30/01/13	1	NA
Green Glades Beach	-37.2768	149.9436	31/01/13	0	NA
Saltwater Creek Beach	-37.1698	150.0031	31/01/13	0	NA
Nullica Beach	-37.0928	149.8726	31/01/13	0	NA
Quarantine Bay	-37.0762	149.8898	31/01/13	0	NA
Cattle Bay	-37.0717	149.8974	31/01/13	0	NA
Aslings Beach	-37.0567	149.9125	31/01/13	0	NA
Long Beach	-36.9753	149.9286	31/01/13	0	NA
Haycock Point	-36.9517	149.9380	31/01/13	0	NA
Lions Beach Pambula	-36.9443	149.9131	31/01/13	0	NA
Pambula Beach South	-36.9406	149.9094	1/02/13	0	NA
Pambula Beach North	-36.8980	149.9150	1/02/13	0	NA
Merimbula Middle Beach	-36.8947	149.9297	1/02/13	1	NA
Merimbula Short Point Beach	-36.8821	149.9301	1/02/13	1	NA
Tura Head	-36.8559	149.9414	1/02/13	1	NA
Bournda Beach South	-36.8140	149.9399	1/02/13	0	NA
Bournda Beach North	-36.7895	149.9584	1/02/13	1	NA
Wallagoot Gap	-36.7876	149.9624	1/02/13	1	NA
Point Ricardo	-37.8056	148.6348	9/05/13	0	0
Corringle Beach	-37.8018	148.4613	9/05/13	0	1
Point Hicks	-37.7895	149.2877	9/05/13	0	1
Bekta Beach	-37.5865	149.7381	9/05/13	0	1
Mallacoota Beach	-37.5686	149.7624	9/05/13	0	0
Whiskey Beach	-39.0122	146.2908	10/05/13	0	0
Seaspray	-38.3674	147.2039	10/05/13	0	0

Paradise Beach	-38.1954	147.4225	10/05/13	0	0
Eastern Beach	-37.8770	148.0158	10/05/13	0	0
Lake Bunga Beach	-37.8693	148.0447	10/05/13	0	0
Nullica Beach	-37.0928	149.8726	11/05/13	0	1
Aslings Beach	-37.0567	149.9125	11/05/13	0	1
Pambula Beach North	-36.8980	149.9150	11/05/13	0	1
Merimbula Middle Beach	-36.8947	149.9297	11/05/13	1	1
Seaspray	-36.8947	149.9297	27/06/13	0	NA
Point Hicks	-36.8947	149.9297	27/06/13	0	NA
Mallacoota Beach	-37.2768	149.9436	27/06/13	0	NA
Green Glades Beach	-37.2768	149.9436	27/06/13	0	NA
Corringle Beach	-35.7579	150.2114	27/06/13	0	NA
Nullica Beach	-37.2768	149.9436	28/06/13	0	NA
Tathra Beach	-36.8947	149.9297	28/06/13	1	NA
Haycock Point	-37.2768	149.9436	28/06/13	0	NA
Merimbula Middle Beach	-37.2768	149.9436	28/06/13	1	NA
Cuttagee Beach	-35.7579	150.2114	28/06/13	1	NA
Handkerchief Beach	-37.2768	149.9436	28/06/13	1	NA
Shoalhaven Heads	-36.8947	149.9297	29/06/13	1	NA
Nullica Beach	-37.2768	149.9436	21/10/13	0	NA
Shoalhaven Heads	-36.8947	149.9297	21/10/13	1	NA
Tathra Beach	-36.8947	149.9297	21/10/13	1	NA
Handkerchief Beach	-37.2768	149.9436	21/10/13	1	NA
Cuttagee Beach	-35.7579	150.2114	21/10/13	1	NA
Merimbula Middle Beach	-37.2768	149.9436	21/10/13	1	NA
Haycock Point	-37.2768	149.9436	21/10/13	0	NA
Point Hicks	-36.8947	149.9297	22/10/13	0	NA
Green Glades Beach	-37.2768	149.9436	22/10/13	0	NA
Mallacoota Beach	-37.2768	149.9436	22/10/13	0	NA
Corringle Beach	-35.7579	150.2114	22/10/13	0	NA
Seaspray	-36.8947	149.9297	23/10/13	0	NA
